# Fungi-Mediated Biotransformation of the Isomeric Forms of the Apocarotenoids Ionone, Damascone and Theaspirane

**DOI:** 10.3390/molecules24010019

**Published:** 2018-12-21

**Authors:** Stefano Serra, Davide De Simeis

**Affiliations:** C.N.R. Istituto di Chimica del Riconoscimento Molecolare, Via Mancinelli 7, 20131 Milano, Italy; dav.biotec01@gmail.com

**Keywords:** biotransformation, oxidation, apocarotenoids, flavours, fungi, ionone, damascone, theaspirane

## Abstract

In this work, we describe a study on the biotransformation of seven natural occurring apocarotenoids by means of eleven selected fungal species. The substrates, namely ionone (α-, β- and γ-isomers), 3,4-dehydroionone, damascone (α- and β-isomers) and theaspirane are relevant flavour and fragrances components. We found that most of the investigated biotransformation reactions afforded oxidized products such as hydroxy- keto- or epoxy-derivatives. On the contrary, the reduction of the keto groups or the reduction of the double bond functional groups were observed only for few substrates, where the reduced products are however formed in minor amount. When starting apocarotenoids are isomers of the same chemical compound (e.g., ionone isomers) their biotransformation can give products very different from each other, depending both on the starting substrate and on the fungal species used. Since the majority of the starting apocarotenoids are often available in natural form and the described products are natural compounds, identified in flavours or fragrances, our biotransformation procedures can be regarded as prospective processes for the preparation of high value olfactory active compounds.

## 1. Introduction

In Nature, the oxidative degradation of the conjugated tetraterpene carotenoids (C_40_) produces a plethora of smaller derivatives, called apocarotenoids [1], which possess a range of different chemical structures and biological activities. Among these natural products, compounds having thirteen carbon atoms in their frameworks are relevant flavours or fragrances and their manufacturing represents an important economic resource for chemical companies [2]. The combination of the great diversity of the carotenoids chemical structures with the different possible degradation pathways, gives rise to a huge number of flavours and fragrances.

**Figure 1:**
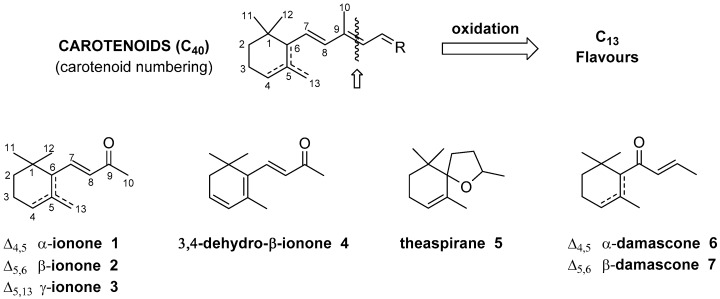
The formation of apocarotenoids through degradation of carotenoids and the seven C_13_ apocarotenoids (compounds **1**–**7**) selected as substrates for the fungal biotransformation investigated in the present study.

It is worth noting that the primary odorous C_13_ apocarotenoids, namely ionone, damascone and theaspirane isomers (Figure 1), are cyclohexene derivatives and the possibility of three different positions of the double bond, the presence of a stereogenic center in position 6 (carotenoids numbering) and the eventual structural rearrangements, can give rise to a large number of isomers. In addition, the latter volatile compounds are often accompanied by structural related apocarotenoids having further oxygen atoms in their chemical framework.

The first consequence of the introduction of a hydroxy- or keto- functional group on these compounds is the decrease of the volatility and the increase of the so-called ‘substantivity’, namely the long lasting odour of a substance having low vapor pressure. The aforementioned compounds have been recognized as components of different natural flavours. For example 3-hydroxy and 3-keto-α-ionone, 4-hydroxy- and 4-keto-β-ionone and hydroxy-β-damascone isomers have been identified in curry tree [3], eucalyptus honey [4], saffron [5], black tea [6] and tobacco [7,8] respectively, whereas 3-keto-theaspirane (also known under the trade name theaspirone) is the character impact compound of the black tea flavour [9].

All these derivatives are present in vegetables in very minute amounts and the extraction is not a viable process for their production. Consequently, they are currently obtained by chemical synthesis and are not commercially available in their natural form. Since flavours possessing ‘natural’ status are usually hundreds times as expensive as their synthetic counterparts, any new procedure that provides these compounds in their high value form can be very profitable. 

In recent years, some new biocatalytic processes have provided a reliable access route to the most common C_13_ apocarotenoids, such as α- and β-ionone. In addition, the genetic engineering of both carotenoids biosynthesis and carotenoids cleavage pathways in the same microbial host [10] has laid the foundation for the large-scale production of the C_13_ apocarotenoids in natural form. 

According to the European and USA legislation, the biotransformation of a natural precursor is a ‘natural method’ of synthesis [11]. Therefore, we singled out the compounds **1**–**7** as prospective natural precursors to be used as starting materials for the biotechnological production of different natural flavours. 

From a biochemical standpoint, both prokaryotes and eukaryotes are able to degrade the carotenoid frameworks. In spite of this fact, only a limited number of biotransformations of the compounds of type **1**–**7** have been reported to date. Although the first description of an ionone isomer biotransformation goes back to 1950, when the oxidation of β-ionone in rabbit was investigated [12], only a limited number of studies on this topic took place in the following years. These researches were based mainly on the fungi and bacteria-mediated chemical transformations whereas the exploitation of some specific oxido-reductases were described only recently. In particular, *Aspergillus niger* [13,14], *Lasiodiplodia theobromae* [15], *Cunninghamella blakesleeana* [16], *Botrytis cinerea* [17], *Aspergillus awamori* [18], *Pleurotus sapidus* [19], *Mortierella isabellina* [20] and different *Streptomyces* strains [21] have proved to be active biocatalysts for the transformation of these kind of compounds. Concerning the use of isolated enzymes or the exploitation of a specific enzymatic activities, both cytochrome P450 monooxygenases [22,23] and engineered whole cell biocatalysts expressing mutant P450 monooxygenases [24], were used for the oxidation of different ionone isomers.

It is worth nothing that the biotransformation of some substrates such as γ-ionone, 3,4-dehydro-β-ionone and α-damascone hasn’t been investigated yet. This paucity of scientific studies is often due to the difficult availability of some apocarotenoids that can be either substrates or products of the biotransformations. For example, γ-ionone is a rare natural isomer of ionone and can be obtained in high isomeric purity only through demanding multistep syntheses [25,26,27,28]. Therefore is not surprising that the fungus-mediated transformation of this compound hasn’t been studied yet. In addition, whole cell biotransformations usually afford very complex mixtures of products whose chemical identification, for example by GC or HPLC analysis, require the availability of the corresponding reference standards. The aforementioned compounds are often not commercially available and have to be prepared by specific and multistep chemical syntheses, thus hampering to perform a proper study on the apocarotenoid’s biotransformation.

Taking advantage of our previous experience on the stereoselective synthesis of ionone and damascone isomers [2,25,26,27,28,29,30,31,32], we decided to set up a comprehensive study on the biotransformation of the seven natural substrates described above by means of eleven selected fungal species belonging to the three more relevant phylums, namely ascomycota, zygomycota and basidiomycota. More specifically, we selected *Aspergillus niger* and *Mortierella isabellina* because these microorganisms have been already used for the biotransformation of some ionone isomers [13,14,20]. The remaining nine strains were singled out among the plethora of the microorganisms described in the literature based on their prospective biotransformation abilities. In effect, *Nigrospora oryzae*, different *Penicillium* species, *Rhizopus stolonifer*, *Curvularia lunata* and *Fusarium culmorum* have been successful employed in the biotransformation of terpenoids and steroids [33,34,35] whereas *Geotrichum candidum* was used for the oxidation of the cyclohexanone derivatives [36]. Finally, we selected also the yeasts *Yarrowia lipolytica* and *Xanthophyllomyces dendrorhous* since they are microorganisms of primary interest in the industrial synthesis/degradation of lipids [37] and carotenoids [38], respectively.

The results obtained by our work, beside confirming and extending those described by some previous researches, give new insights on the ability of fungi in the biotransformation of apocarotenoids, establishing their prospective utility for flavour production.

## 2. Results and Discussion

As described in the introduction, each one of the selected apocarotenoid isomers was incubated with a growing culture of each one of the eleven fungal strains. After a defined period of time (see Experimental Section) the crude biotransformation mixtures were derivatized (by acetylation) and analysed by GC-MS. To this end, several reference standard compounds were prepared by chemical synthesis and then were used for the unambiguous identification of the compounds formed in the biotransformation experiments. In spite of our efforts, a number of these derivatives were not identified. Therefore, in order to spot the most relevant biochemical transformations that each fungal strain is able to perform, we carried out the chromatographic isolation of the unknown metabolites that were formed in relevant amount or that make up of the main part of the transformed derivatives. The structures of these compounds were then elucidated through their comprehensive chemical characterization. More specifically, the combined use of ^1^H-NMR, ^13^C-NMR, DEPT experiments, GC-MS and ESI-MS spectroscopy allowed us to identify some compounds that haven’t been correlated with any biotransformation experiment yet or that haven’t been described in the literature until now.

We first investigated the transformation of the ionone isomers **1**–**3**. Although only α- and β-ionone are very common in Nature, we deemed that it would be very interesting to also study the reactivity of the rare γ-isomer. In effect, even if the latter three compounds differ only for the position of the cyclohexene double bond, they possess very different reactivity from each other. Therefore, the identification of the derivatives obtained through biotransformation can help in understanding the chemical processes involved in these fungi-mediated reactions. 

**Figure 2:**
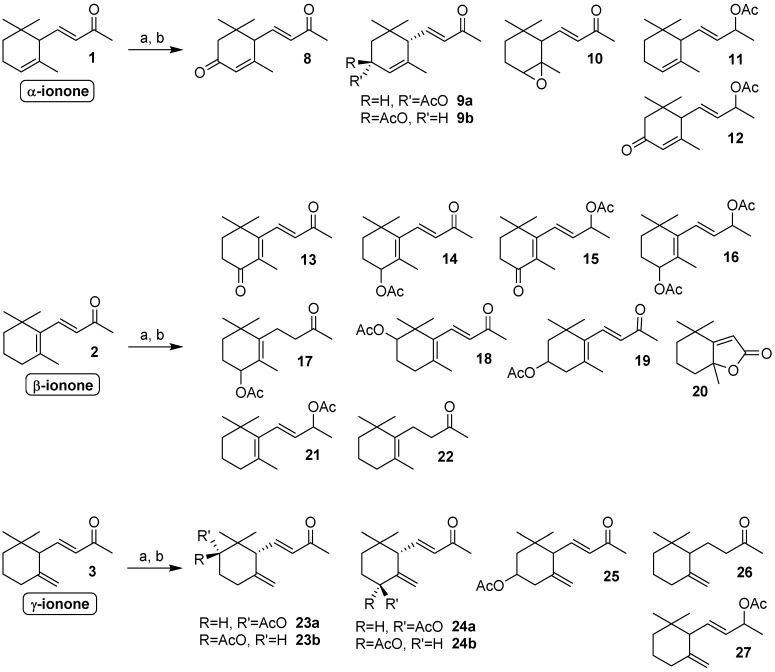
Compounds obtained through biotransformation of α-, β- and γ-ionone by means of the fungal strains employed in the present study. *Reagents and conditions*: (**a**) fungal strain, malt extract medium (MEM), aerobic conditions, 140 rpm, 20 or 25 °C, 8–20 days; (**b**) Ac_2_O/pyridine (Py), 4-dimethylaminopyridine (DMAP) catalyst, room temperature (rt), 6 h.

As collectively described in Figure 2, the main part of the identified metabolites are the result of four different biochemical reactions, namely the oxidation of the methylene functional groups, the reduction of the conjugated double bond, the epoxidation of the 4,5-double bond and the reduction of the keto group. The investigated fungal strains are able to perform both single step reactions and sequential multi-steps transformations, in turn deriving from the combination of the aforementioned four chemical reactions. 

Overall, ionone isomers **1**–**3** were converted into derivatives **8**–**27** in relative amounts described in Table 1. A thorough perusal of these data shows considerable differences in the reactivity of the three isomers. α-Ionone **1** was oxidized almost exclusively at the activated allylic methylene functional group, with the exception of *Geotrichum candidum* that is completely inactive and of *Curvularia lunata* and *Fusarium culmorum* that are also able to oxidize the 4,5-double bond to give the corresponding epoxy-ionone **10**. The relative ratio of the obtained 3-keto-α-ionone **8** and of the diastereoisomeric 3-hydroxy-α-ionones **9a** and **9b** changes significantly based on the fungal strain used. Overall, the microorganisms that better performed the oxidation of **1** were *Aspergillus niger*, *Nigrospora oryzae* and *Fusarium culmorum*.

The global amount of the 3-oxidized metabolites obtained using the latter fungal strains took account of the 74%, 69% and 58% of the crude biotransformation mixture, respectively. On the contrary, none of the tested strains showed notable reductive activity on α-ionone, as confirmed by the modest formation of derivatives **11** and **12**.

A more complex outcome were observed when β-ionone **2** was used as substrate. In this case, even if the latter ketone was oxidized mainly at the activated allylic methylene functional group (compounds **13**–**17**), the biotransformations also afforded the 2-hydroxy-β-ionone derivative **18** (*Aspergillus niger* and *Penicillium corylophilum*) and a little amount of 3-hydroxy-β-ionone derivative **19** (*Penicillium roqueforti*). In addition, the partial oxidation of the ionone side chain is also possible, as shown by the detection of trace (1%) of dihydroactinodiolide **20** in the biotransformation mixture of *Geotrichum candidum*. Interestingly, all the tested fungal strains left unaffected the C(13) methyl group of both α- and β-ionone isomers. In effect, we observed neither the formation of 13-hydroxy-derivatives nor the presence of the epoxy-megastigmaen-9-one isomers that can arise from the intramolecular 1,4-addition of the hydroxy-group to the conjugated double bond [31].

On the contrary, the reduction of the conjugated double bond and of the keto group are chemical transformations of major significance in fungal β-ionone biotransformation. The abovementioned reactions can proceed also on intermediates deriving from the oxidation of the activated position 4 of the β-ionone framework, affording a number of oxidized-reduced derivatives (**15**–**17**) besides compounds obtained by simple reduction (i.e., **21** and **22**).

Completely different results were observed for the biotransformation of γ-ionone where the oxidation of the allyl methylene group, to afford derivative **24**, appeared to be a path of minor relevance. On the contrary, the oxidation of the positions 2 and 3 of the latter ionone isomer was efficiently performed by *Aspergillus niger* that produced compounds **23a** and **25** in high yield. *Penicillium corylophilum* is also able to convert γ-ionone into the derivative **23a** but for this microorganism, as well as for *Yarrowia lipolytica*, the reductive steps are more relevant than the oxidative ones. In effect, the reduction of the conjugated double bond and of the carbonyl functional group afforded the compound **26** and **27**, respectively. It is worth noting that *Aspergillus niger*, *Penicillium roqueforti* and *Penicillium corylophilum* produced diastereoselectively *cis*-2-hydroxy-γ-ionone (**23a**) and none of the microorganisms tested afforded its diastereoisomer, namely *trans*-2-hydroxy-γ-ionone (**23b**). Differently, the fungi *Nigrospora oryzae*, *Curvularia lunata* and *Fusarium culmorum* transformed efficiently γ-ionone, but the main part of the biotransformation reaction consisted in a mixture of unknown compounds, most likely deriving by the extensive oxidative degradation of the ionone framework.

**Figure 3:**
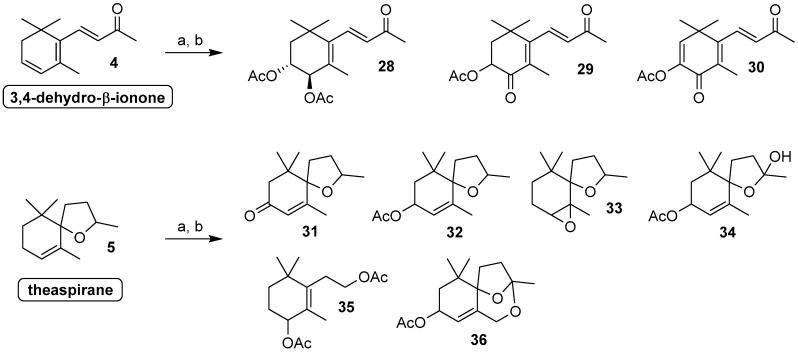
Compounds obtained through biotransformation of 3,4-dehydro-β-ionone and theaspirane by means of the fungal strains employed in the present study. *Reagents and conditions*: (**a**) fungal strain, MEM, aerobic conditions, 140 rpm, 20 or 25 °C, 14–20 days; (**b**) Ac_2_O/Py, DMAP catalyst, rt, 6 h.

The second group of the investigated apocarotenoids regards 3,4-dehydro-β-ionone (**4**) and theaspirane (**5**, Figure 3).

Although these compounds have different chemical structures, they both showed high reactivity and the results of their biotransformation experiments were described together. More specifically, we observed that all the investigated fungal strains were not able to oxidize the allylic positions of compound **4**. We identified as biotransformation products only compounds **28**–**30** (Table 2). These three ionone derivatives originated from the oxidation of the conjugated diene functional group. Most likely the first step is the epoxidation of the 3,4-double bond followed by the opening of the oxirane ring by addition of a molecule of water. This two step mechanism could justify the formation of the compound **28**, possessing *trans* relative configuration of the two contiguous hydroxy groups, as the major diastereoisomer. In addition, the following oxidation of the alcohol functional groups gave rise to the keto derivatives. More specifically, the oxidation at position 4 or at both position 4 and 3 gave compounds **29** and **30**, respectively. 

Otherwise, when a spirocyclic ether group replaces the conjugated carbonyl group, our selected fungal strains become able to oxidize the theaspirane framework both at the allylic positions and at the methine carbon linked to the ether oxygen atom. Overall, the main part of the compounds obtained by biotransformation of **5** derive from allylic oxidation. In particular, the experiments performed using *Aspergillus niger*, *Rhizopus stolonifer* and *Fusarium culmorum* showed a total content of the compounds **31** and **32** that accounts for at least 60% of the reaction mixtures. This result is noteworthy as theaspirone **31** is a relevant natural flavour and the corresponding alcohol (**32** is the acetylated derivative) can be regarded as its direct precursor. In effect, the preparation of **31** in natural form could be possible by means of oxidation of the allyl alcohol, for example using a biocatalytic transformation involving alcohol dehydrogenases.

Jointly with allylic oxidation, we observed also the oxidation of the ether functional group. Hence, the latter moiety was transformed into the hemiketal and ketal groups as well in a completely rearranged framework, as demonstrated by the isolation and characterization of compounds **34**, **36** and **35**, respectively. Most likely, the latter derivatives are the result of a multistep oxidation reaction. The transformation of the allyl methylene and methyl groups proceeds faster than the oxidation of ether functional group, which is finally converted into a hemiketal functional group. Accordingly, *Nigrospora oryzae* and *Mortierella isabellina* completely oxidized theaspirane (**5**) to give two biotransformation mixtures containing 28% and 50% of compound **34**, respectively. It is worth mentioning that the latter compound is the direct precursor of 8,9-dehydrotheaspirone [32], a relevant apocarotenoid flavour identified in white-fleshed nectarines [39]. 

The biotransformation experiments performed using *Curvularia lunata* gave results of more complex interpretation affording a plethora (60% of the mixture) of undetermined compounds beside the 3%, 8%, 12% and 9% of derivatives **32**, **34**, **35** and **36**, respectively. Compound **35** could result from a multistep transformation comprising both of oxidation and transposition reactions. The C(9) oxidation is responsible for the formation of the hemiketal functional group that is in equilibrium with the less stable open hydroxy-ketone form. The Baeyer-Villiger oxidation of the latter ketone functional group could explain the formation of the primary alcohol acetate moiety whereas the 1,3-allyl transposition of the tertiary hydroxy group could take account of the unexpected position of the C(4) acetate group in compound **35**. Similarly, the formation of compound **36** in *Curvularia lunata*–mediated biotransformation could be explained by the hydroxylation of the carbons placed in position 3, 9 and 13 of the ionone framework.

The subsequent reaction of the obtained primary alcohol group with the hemiketal functional group should afford the more thermodynamically stable tricyclic ketal **36**. It is worth nothing that a natural compound having an identical ketal structure, but devoid of the hydroxy group at the C(4), is an aroma component of quince brandy [40]. The comparison of the NMR data measured for **36** with those reported for the natural flavour, allowed us to assign the above described chemical structure suggesting a new synthetic approach to this chemical framework by fungal biotransformation of theaspirane. 

**Figure 4:**
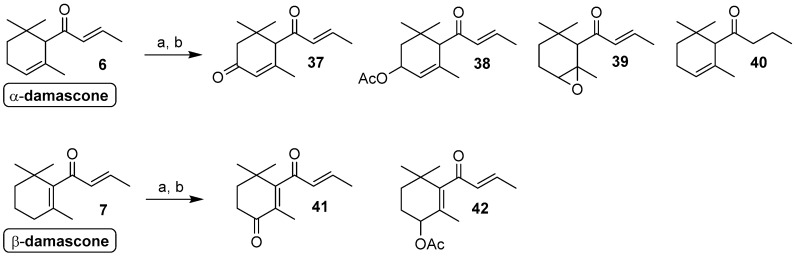
Compounds obtained through biotransformation of α- and β-damascone by means of the fungal strains employed in the present study. *Reagents and conditions*: (**a**) fungal strain, MEM, aerobic conditions, 140 rpm, 20–25 °C, 14–20 days; (**b**) Ac_2_O/Py, DMAP catalyst, rt, 6 h.

A completely different reactivity was observed in the biotransformation of the α- and β-damascone isomers **6** and **7** (Figure 4). The latter apocarotenoids are isomers of α- and β-ionone respectively, as each one damascone isomer is interconvertible into the corresponding ionone isomer by 1,3-shift of the enone moiety.

Despite the structural similarity of these compounds, none of the investigated fungal strains was able to reduce the carbonyl functional group present in the damascone framework. In addition, the fungi-mediated oxidative transformations of damascone isomers are restricted mainly to the allyl methylene functional groups, as indicated by the formation of derivative **37** and **38** from α-damascone and derivatives **41** and **42** from β-damascone (Table 3). The other position of the damascone framework were unaffected with the exception of the 4,5-double bond that was oxidized by both *Mortierella isabellina* and *Xanthophyllomyces dendrorhous* to produce a very minor amount of the epoxy-α-damascone **39**. Similarly, we did not record any reductive reactions with the exception of the conjugated double bond of the α-damascone isomer that was reduced by *Penicillium corylophilum* to give a small amount of compound **40**. 

Overall, using the described fungal strains, we observed that the damascone isomers are less reactive than the corresponding ionone isomers. This effect is more pronounced for the β-isomer where only *Nigrospora oryzae* and *Fusarium culmorum* produced a significant amount of the corresponding 4-hydroxydamascone, identified as acetyl derivative **42** (23 and 22%, respectively). 

Our results seem in contrast to those reported in a recent study on the fungal biotransformation of β-damascone [20], where a different *Mortierella isabellina* strain provides 4-hydroxydamascone in a much higher yield. The recorded differences between these experimental data could be justified considering both the substrate concentrations and the different biocatalytic activity among fungal strains belonging to the same species.

In particular the substrate concentration seem to be the most relevant factor as apocarotenoid derivatives show significant toxicity for many fungal strains and high concentration of these compounds could inhibit their growth as well as their biocatalytic activity. We performed all the investigated biotransformation experiments using a substrate concentration of about 2.5 g/L whereas, in the above-mentioned work, the β-damascone concentration was set to 0.1 g/L. Our choice is justified by the need of devising a preparative protocol for fungi-mediated ionone biotransformation. Since the synthesis of this kind of flavours, in natural form, can show industrial significance only working with substrate concentrations superior to 1 g/L, we set the above indicated concentration for all experiments. As a consequence, it is reasonable that our selected fungal strains could have transformed better both damascone as well as ionone and theaspirane isomers if they have been used in lower concentration. 

## 3. Materials and Methods

### 3.1. Materials and General Methods

All air and moisture sensitive reactions were carried out using dry solvents and under a static atmosphere of nitrogen. All solvents and reagents were of commercial quality and were purchased from Sigma-Aldrich (St. Louis, MO, USA). A large number of reference standard compounds were synthesized in our laboratory and were used for the unambiguous identification of the compounds formed in the biotransformation experiments. α-Ionone, γ-ionone and α-damascone were used in racemic form. Commercial theaspirane consists of an equimolar mixture of racemic diastereoisomers. γ-Ionone (**3**) and 3,4-dehydro-β-ionone (**4**) were prepared starting from α-ionone, according to the procedures previously described by us [26,27,28].

The keto derivatives: 3-keto-α-ionone (**8**), 4-keto-β-ionone (**13**), 3-keto-α-damascone (**37**), 4-keto-β-damascone (**41**), theaspirone (**31**), 3-keto-α-ionol acetate (**12**) and 4-keto-β-ionol acetate (**15**) were prepared by oxidation of α-ionone, β-ionone, α-damascone, β-damascone, theaspirane, α-ionol acetate (**11**) and β-ionol acetate (**21**), respectively. The oxidation reactions were performed using TBHP/MnO_2_ as oxidant according to our previously reported procedure [41]. 

The diastereoisomeric forms of 4,5-epoxy-α-ionone (**10**), 4,5-epoxy-α-damascone (**39**), 4,5-epoxy-theaspirane (**33**) as well as 5,6-epoxy-β-ionone and 5,6-epoxy-β-damascone were prepared by epoxidation of α-ionone, α-damascone, theaspirane, β-ionone and β-damascone, respectively, using *m*-chloroperbenzoic acid and CH_2_Cl_2_ as solvent. 

α-7,8-Dihydroionones, β-7,8-dihydroionone (**22**) and γ-7,8-dihydroionone (**26**) were prepared by reduction of α-, β- and γ-ionone respectively, using hydrogen and Ni Raney as catalyst for α-ionone [42] and Bu_3_SnH and (Ph_3_P)_2_PdCl_2_ as catalyst for β- and γ-ionone [25,26]. α-8,9-Dihydrodamascone (**40**) was prepared by reduction of α-damascone using NaBH_4_ in methanol. β-8,9-Dihydrodamascone was prepared by addition of propylmagnesium bromide to β-cyclocitral followed by oxidation of the resulting carbinol using Dess-Martin periodinane [43]. 

β-Ionol acetate (**21**, racemic), α-ionol acetate (**11**, as a mixture of two racemic diastereoisomers), γ-ionol acetates (**27**, as a mixture of two racemic diastereoisomers) and 3-acetoxy-theaspirane (**32**) (as a mixture of four racemic diastereoisomers) were prepared by chemical acetylation (Ac_2_O/Py) of the corresponding alcohols, which were in turn obtained through the reduction of α-, β-, γ-ionone and theaspirone, respectively, using NaBH_4_ in methanol.

*cis*-2-Acetoxy-α-ionone, and *cis*-2-acetoxy-γ-ionone (**23a**) were prepared starting from 2,8,8-trimethyl-6-oxabicyclo[3.2.1]oct-2-en-7-one and 8,8-dimethyl-2-methylene-6-oxabicyclo[3.2.1]octan-7-one (kaharana lactone) respectively, according to the synthetic procedure developed by Audran [44]. The latter lactones were in turn prepared from racemic *cis*-2-hydroxy-α-cyclogeraniol and *cis*-2-hydroxy-γ-cyclogeraniol [45] by oxidation using BAIB and TEMPO as catalyst. In addition, the partial reduction of the two isomeric lactones afforded the corresponding lactols, whose condensation with acetone [46] followed by acetylation (Ac_2_O/Py) of the crude reaction mixtures, gave the *cis*/*trans* mixtures of acetoxy-α-ionone and acetoxy-γ-ionone, respectively, that were used as GC-MS reference standards for the identification of the corresponding *trans* isomers. 

Racemic 2-acetoxy-β-ionone (**18**) was prepared starting from 2-hydroxy-β-cyclogeraniol [45] by selective oxidation of the primary alcohol functional group by MnO_2_ in CHCl_3_, condensation with acetone [46] and acetylation (Ac_2_O/Py) of the obtained hydroxy-ionone derivative.

Samples of 3-acetoxy-α-ionone (**9**) (2:1 *cis*/*trans* mixture), of 4-acetoxy-γ-ionone (**24**) (4:1 *cis*/*trans* mixture) and of 3-acetoxy-β-ionone (**19**) were prepared starting from α-ionone according to the procedure described by Tu [47], by Serra [42] and Khachik [48], respectively. A sample of 3-acetoxy-α-damascone (**38**) (1:1 *cis*/*trans* mixture) was prepared starting from ethyl 3-hydroxy-α-cyclogeraniate according to the procedure described by Takei [49].

4-Acetoxy-β-ionone (**14**) and 4-acetoxy-β-damascone (**42**) were prepared starting from 4,5-epoxy-α-ionone and 4,5-epoxy-α-damascone, respectively, by means of NaOMe mediated transposition followed by chemical acetylation (Ac_2_O/Py) of the obtained allyl alcohols. A sample of 4-acetoxy-β-ionol acetate (**16**) (1:1 mixture of diastereoisomers) was prepared from 4-keto-β-ionol acetate by reduction with NaBH_4_ in methanol followed by chemical acetylation (Ac_2_O/Py).

A sample of 4-acetoxy-β-7,8-dihydroionone acetate (**17**) was prepared from 4-hydroxy-β-ionone by reduction with Ph_3_SiH followed by chemical acetylation (Ac_2_O/Py), according to the procedure described by Pascual [50].

3,4-Diacetoxy-β-ionone (**28**) (*cis*/*trans* mixture), was prepared from 3,4-dehydro-β-ionone according to the procedure described by Buschor [51]. The oxidation of 4-keto-β-ionone with IBDA in methanol [52] afforded 3-hydroxy-4-keto-β-ionone that was further oxidated using oxygen in presence of *t*BuOK [53] to give 3,4-diketo-β-ionone. The acetylation (Ac_2_O/Py) of the latter two compounds afforded 3-acetoxy-4-keto-β-ionone (**29**) and 3-acetoxy-4-keto-2,3-dehydro-β-ionone (**30**). Racemic dihydroactinodiolide (**20**) and 2-hydroxy-2,6,10,10-tetramethyl-1-oxaspiro[4.5]dec-6-en-8-yl acetate (**34**) were prepared as described previously [32,54]. 

A comprehensive characterization of the above described reference standards is reported in the Supplementary Materials section.

### 3.2. Analytical Methods and Characterization of the Products Deriving from the Biotransformation Experiments

The crude biotransformation mixtures obtained according to the procedures described below were then acetylated by treatment with pyridine/acetic anhydride (2 mL of a 2:1 mixture) and catalytic DMAP (10 mg) for 6 hours at rt. The obtained acetylated mixture was analyzed by GC-MS. The compounds whose chemical structure couldn’t be assigned only by GC-MS analysis were isolated from the biotransformation mixtures by means of chromatographic separation and then characterized by NMR analysis and GC-MS or ESI-MS analysis.

^1^H- and ^13^C-NMR spectra and DEPT experiments were recorded/performed at 400, 100 and 100 MHz, respectively, in CDCl_3_ solutions at rt using an AC-400 spectrometer (Bruker, Billerica, MA, USA); ^13^C spectra are proton decoupled; chemical shifts in ppm rel to internal SiMe_4_ (=0 ppm).

TLC: silica gel *60 F_254_* plates (Merck, Kenilworth, NJ, USA). Column chromatography: silica gel. 

Melting points were measured on a Reichert apparatus, equipped with a Reichert microscope, and are uncorrected.

Mass spectrum were recorded on a ESQUIRE 3000 PLUS spectrometer equipped with an ESI detector (Bruker, Billerica, MA, USA) or by GC-MS analyses.

GC-MS analyses: *HP-6890* gas chromatograph equipped with a 5973 mass detector, using a HP-5MS column (30 m × 0.25 mm, 0.25 μm film thickness; Hewlett Packard, Palo Alto, CA, USA) with the following temp. program: 60° (1 min)—6°/min—150° (1 min)—12°/min—280° (5 min); carrier gas, He; constant flow 1 mL/min; split ratio, 1/30; *t*_R_ given in min: *t*_R_(**1**) 16.23, *t*_R_(**2**) 17.57, *t*_R_(**3**) 16.53, *t*_R_(**4**) 17.53, *t*_R_(**5**) 13.40 and 13.77, *t*_R_(**6**) 15.44, *t*_R_(**7**) 15.91, *t*_R_(**8**) 20.42, *t*_R_(**9a**) 21.41, *t*_R_(**9b**) 21.61, *t*_R_(**10**) 18.63 and 18.72, *t*_R_(**11**) 17.78, *t*_R_(**12**) 21.52, *t*_R_(**13**) 20.61, *t*_R_(**14**) 21.82, *t*_R_(**15**) 22.03, *t*_R_(**16**) 22.56, *t*_R_(**17**) 21.59, *t*_R_(**18**) 22.18, *t*_R_(**19**) 22.23, *t*_R_(**20**) 18.61, *t*_R_(**21**) 18.55, *t*_R_(**22**) 16.51, *t*_R_(**23a**) 21.51, *t*_R_(**24a**) 21.32, *t*_R_(**24b**) 21.60, *t*_R_(**25**) 21.79, *t*_R_(**26**) 15.67, *t*_R_(**27**) 17.47, 17.58, 18.01 and 18.08, *t*_R_(**28**) 24.10, *t*_R_(**29**) 23.75, *t*_R_(**30**) 23.92, *t*_R_(**31**) 19.02 and 19.19, *t*_R_(**32**) 20.17, 20.33, 20.50 and 20.61, *t*_R_(**33**) 15.14 and 15.50, *t*_R_(**34**) 19.37 and 19.67, *t*_R_(**35**) 21.78, *t*_R_(**36**) 21.64, *t*_R_(**37**) 20.15, *t*_R_(**38**) 21.17 and 21.30, *t*_R_(**39**) 17.84, *t*_R_(**40**) 14.62, *t*_R_(**41**) 20.91, *t*_R_(**42**) 20.93, *t*_R_(7,8-dihydro-α-ionone) 15.95, *t*_R_(7,8-dihydro-β-damascone) 20.95, *t*_R_(*cis*-2-acetoxy-α-ionone) 21.42, *t*_R_(*trans*-2-acetoxy-α-ionone) 21.36.

### 3.3. Microorganisms and Biotransformation Experiments

Geotrichum candidum (DSM 10452), Yarrowia lipolytica (DSM 8218), Rhizopus stolonifer (DSM 855), Xanthophyllomyces dendrorhous (DMS 5626), Curvularia lunata (CBS 215.54), Mortierella isabellina (CBS 167.60), Aspergillus niger (CBS 626.26) were purchased from the DSMZ (Braunschweig, Germany) or CBS-KNAW (Utrecht, The Netherlands) collections. 

*Penicillium corylophilum* (MUT 5838), *Nigrospora oryzae* (MUT 5844), *Penicillium roqueforti* (MUT 5856) and *Fusarium culmorum* (MUT 5855) were isolated as axenic cultures in our laboratory, then identified by the Mycotheca Universitatis Taurinensis (MUT) of the University of Turin and finally deposited in the same institution under the collection number given in brackets.

All the biotransformations were carried out in triplicate and the presented results are the average of three experimental runs.

#### 3.3.1. Representative Procedures for Biotransformations

The experimental conditions used for the biotransformations are based on the type of microorganism used. Here is described a general procedure depending on the different morphological features regarding the various active grow mycelia. The main ones could be classified in yeast-shape mycelia (*Xanthophyllomyces dendrorhous*, *Geotrichum candidum* and *Yarrowia lipolytica*) and spore-forming mycelia (*Aspergillus niger*, *Rhizopus stolonifer*, *Curvularia lunata*, *Penicillium corylophilum*, *Nigrospora oryzae*, *Penicillium roqueforti*, *Mortierella isabellina* and *Fusarium culmorum*). In the first case, a small amount of the active mycelia grew previously in a petri dish, was suspend in 1 mL of sterile water and then inoculated in a 100 mL conical Pyrex flask containing 40 mL of Malt Extract Medium (MEM) for 2 days at 25 °C and 140 rpm (with exception of *Xanthophyllomyces dendrorhous* that was grown at 20 °C). After this period, the cells were centrifuged 3 minutes, (rt, 3220·*g*) and collected removing the media. The cells (approx. 600 mg wet-weight) were suspended in 3 mL of sterile water than 350 µL of the same suspension were used for inoculating each biotransformation flask containing 40 mL of MEM. In order to ensure aerobic conditions, the flasks were sealed with cellulose plugs. The microorganism was leave to growth for 2 days and then was treated with a solution of 100 mg of substrate dissolved in 400 µL of DMSO. Generally, after 14 days from the substrate injection using the growing condition described above, the reaction media was filtered under vacuum through a celite pad then was extracted 3 times with ethyl acetate. The organic phase was separated, dried on Na_2_SO_4_ and the solvent removed at reduced pressure to give the crude biotransformation mixture.

In the case of the spore-forming mycelia, the spore were collected from a sporulated surface cultures and suspended in 3 mL of sterile water. After that, 350 µL of the same suspension were used for inoculating each biotransformation flask containing 40 mL of MEM. The subsequent steps are the same of that described previously. The unique exception was carried out for *Penicillium corylophilum*, in the case of γ-ionone. The toxicity of the compound forced us to keep its concentration lower than the others (7.5 mM) and to block the biotransformation earlier (8 days). After this period, the most important products are degraded. In the case of *Fusarium culmorum* the biotransformation was blocked after 20 days instead of 14 days because the activity of the fungus did not stop in the prefixed time.

#### 3.3.2. Preparative Biotransformations and Chemical Characterization of Compounds **23a**, **25**, **28**, **35** and **36**

For the main part of the strains tested, the GC-MS analysis of the crude biotransformation mixtures indicated the presence of different compounds whose chemical structures could not be assigned only on the basis of our reference standards. The unidentified peaks taking account of less than 5% of the overall percentage of the compounds obtained by biotransformation were collectively indicated as ‘not determined’. Otherwise, compounds **23a**, **25**, **28**, **35** and **36** were isolated from the fermentation broths by extraction and chromatographic separation and then submitted to chemical characterization. Different reasons prompted us to undertake the isolation procedure. First of all, these compounds were a relevant part of the biotransformation mixture and their MS fragmentations clearly indicated a chemical structure deriving from the corresponding starting materials. The compounds **25** was not one of the references standard available from our laboratory. Hence, we identified this compound only after its isolation and chemical characterization. Both the diastereoisomeric forms of the compound **25** have been described in the literature [55] but only low resolution ^1^H-NMR data was reported. Therefore, we were not able to assign the relative configuration to the compound obtained by biotransformation. Differently, compound **36** is completely new and its analytic data has not described yet. Concerning compound **28**, we observed that its diastereoisomeric forms (*trans* and *cis* isomers) have the same retention time by GC-MS analysis. As a consequence, the isolation of compound **28** from the biotransformation mixture followed by its NMR analysis was necessary in order to understand what was the main isomer formed. Finally, the case of compound **35** is singular. It is the only compound obtained by biotransformation that was formed through a Baeyer-Villiger oxidation. Consequently, acetate **35** was completely unexpected and the proper reference standard was not synthesized.

Hereafter we reported the procedure for the preparative biotransformation experiments allowing the isolation of compounds **23a**, **25**, **28**, **35** and **36** as well as their main analytic data.

According to the procedure described before for the preparation of the inoculum of spore forming mycelia, *Aspergillus niger*, *Nigrospora oryzae* and *Curvularia lunata*, were inoculated in three 1 L conical pyrex flasks containing 400 mL of MEM. The microorganisms were left to grow at 25 °C and 140 rpm for 2 days. Hence the cultures of *Aspergillus niger*, *Nigrospora oryzae* and *Curvularia lunata*, were treated with a solution of 1 g of γ-ionone, 3,4-dehydro-β-ionone and theaspirane, respectively, each one dissolved in 3 mL of DMSO. After 14 days from the substrate injection, using the growing condition described above, the reaction media was filtered through a celite pad, the filter was washed with ethyl acetate and the filtrate was extracted 3 times with the same solvent. The combined organic phases were separated, were washed with brine, dried on Na_2_SO_4_ and the solvent was removed under reduced pressure. The residue was then acetylated by treatment with pyridine/acetic anhydride (10 mL of a 2:1 mixture) and catalytic DMAP (10 mg) for 6 h at rt. The acetylating mixture (Py/Ac_2_O) was then removed under reduced pressure and the resulting oil was purified by chromatography using *n*-hexane/AcOEt mixture as eluent.

The biotransformation of γ-ionone performed using *Aspergillus niger* allowed isolating 0.21 g (18% yield) of compound **23a** and 0.36 g (28% yield) of compound **25** as a single diastereoisomeric form (configuration not determined):

*cis*-2-Hydroxy-γ-ionone acetate (**23a**) = *(1SR,3RS)-2,2-Dimethyl-4-methylene-3-((E)-3-oxobut-1-en-1-yl)cyclohexyl acetate*. ^1^H-NMR: δ = 6.97 (dd, *J* = 15.8, 9.9 Hz, 1H), 6.10 (d, *J* = 15.8 Hz, 1H), 4.88 (s, 1H), 4.74 (dd, *J* = 9.2, 4.0 Hz, 1H), 4.61 (s, 1H), 2.65 (d, *J* = 9.9 Hz, 1H), 2.41 (dt, *J* = 14.0, 5.4 Hz, 1H), 2.37–2.05 (m, 1H) 2.28 (s, 3H), 2.07 (s, 3H), 1.93–1.83 (m, 1H), 1.72–1.57 (m, 1H), 0.91 (s, 6H). ^13^C-NMR δ = 197.9 (C), 170.4 (C), 146.0 (C), 145.5 (CH), 132.9 (CH) 111.1 (CH_2_), 77.8 (CH), 55.9 (CH), 39.0 (C), 31.2 (CH_2_), 27.6 (CH_2_), 27.3 (Me), 26.2 (Me), 21.1 (Me), 17.8 (Me). GC-MS (EI): *m*/*z* (%) = 250 [M^+^] (12), 235 [M^+^ − Me] (1), 208 (13), 190 (45), 175 (40), 165 (36), 147 (100), 131 (23), 122 (39), 109 (96), 91 (34), 79 (35), 71 (12).

3-Hydroxy-γ-ionone acetate (**25**) = *(E)-3,3-Dimethyl-5-methylene-4-(3-oxobut-1-en-1-yl)cyclohexyl acetate*. ^1^H-NMR: δ = 6.82 (dd, *J* = 15.8, 10.1 Hz, 1H), 6.13 (d, *J* = 15.8 Hz, 1H), 4.97 (s, 1H), 4.95–4.86 (m, 1H), 4.63 (s, 1H), 2,73 (dd, *J* = 12.6, 4.9 Hz, 1H), 2.58 (d, *J* = 10.1 Hz, 1H), 2.29 (s, 3H), 2.15–2.01 (m, 1H), 2.03 (s, 3H), 1.85 (dd, *J* = 12.6, 4.5 Hz, 1H), 1.45 (t, *J* = 12.1 Hz, 1H), 0.95 (s, 3H), 0.92 (s, 3H). ^13^C-NMR: δ = 197.8 (C), 170.3 (C), 145.3 (CH), 144.5 (C), 134.0 (CH), 112.5 (CH_2_), 69.8 (CH), 56.0 (CH), 45.5 (CH_2_), 41.1 (CH_2_), 35.8 (C), 30.4 (Me), 27.3 (Me), 21.4 (Me), 21.3 (Me). GC-MS (EI): *m*/*z* (%) = 250 [M^+^] (<1), 235 [M^+^ − Me] (<1), 190 (28), 175 (29), 157 (14), 147 (100), 131 (32), 119 (20), 105 (39), 91 (27), 79 (15), 69 (14), 55 (8).

The biotransformation of 3,4-dehydro-β-ionone performed using *Nigrospora oryzae* allowed isolating 0.56 g (34% yield) of compound **28** as a 5:1 mixture of *trans*/*cis* isomers:

*trans*-3,4-Dihydroxy-β-ionone diacetate (**28**) = *(1RS,2RS)-3,5,5-Trimethyl-4-((E)-3-oxobut-1-en-1-yl)cyclohex-3-ene-1,2-diyl diacetate*. ^1^H-NMR: δ = 7.10 (dq, *J* = 16.4, 0.9 Hz, 1H), 6.11 (d, *J* = 16.4 Hz, 1H), 5.51 (d, *J* = 7.8 Hz, 1H), 5.17–5.09 (m, 1H), 2.28 (s, 3H), 2.06 (s, 3H), 2.00 (s, 3H), 1.88–1.66 (m, 2H), 1.62 (br s, 3H), 1.17 (s, 3H), 1.07 (s, 3H). ^13^C-NMR: δ = 197.8 (C), 170.7 (C), 170.4 (C), 141.0 (CH), 140.2 (C), 133.7 (CH), 128.5 (C), 74.7 (CH), 70.3 (CH), 41.4 (CH_2_), 36.4 (C), 29.7 (Me), 27.7 (Me), 27.5 (Me), 21.1 (Me), 20.8 (Me), 16.5 (Me). MS (ESI): 331.2 (M^+^ + Na).

The biotransformation of theaspirane performed using *Curvularia lunata* allowed isolating 95 mg (7% yield) of compound **35** and 65 mg (5% yield) of compound **36**:

*2-(3-Acetoxy-2,6,6-trimethylcyclohex-1-en-1-yl)ethyl acetate* (**35**). ^1^H-NMR: δ = 5.13 (t, *J* = 4.6 Hz, 1H), 4.05 (dd, *J* = 9.4, 7.5 Hz, 2H), 2.46–2.37 (m, 2H), 2.06 (br s, 6H), 1.92–1.79 (m, 1H), 1.74–1.52 (m, 2H), 1.66 (s, 3H), 1.44–1.33 (m, 1H), 1.08 (s, 3H), 1.00 (s, 3H). ^13^C-NMR: δ = 171.0 (C), 171.0 (C), 139.5 (C), 128.2 (C), 72.3 (CH), 63.3 (CH_2_), 35.1 (C), 34.6 (CH_2_), 28.2 (Me), 28.0 (CH_2_), 26.8 (Me), 25.3 (CH_2_), 21.3 (Me), 21.0 (Me), 16.8 (Me). GC-MS (EI): *m*/*z* (%) = 268 [M^+^] (<1), 226 (13), 208 [M^+^ − AcOH] (16), 166 (40), 148 (17), 133 (100), 120 (28), 110 (36), 91 (16), 79 (8).

*3,6,6-Trimethyl-1,4,5,6,7,8-hexahydro-3H-3,5a-epoxybenzo[c]oxepin-8-yl acetate* (**36**). ^1^H-NMR: δ = 5.43–5.36 (m, 1H), 5.31 (s, 1H), 4.49 (dt, *J* = 14.2, 2.1 Hz, 1H), 4.20 (d, *J* = 14.2 Hz, 1H), 2.30–1.40 (m, 6H), 2.02 (s, 3H), 1.47 (s, 3H), 1.11 (s, 3H), 1.01 (s, 3H). ^13^C-NMR: δ = 170.5 (C), 138.7 (C), 116.3 (CH), 105.3 (C), 86.0 (C), 68.4 (CH), 63.7 (CH_2_), 40.7 (CH_2_), 35.3 (C), 34.3 (CH_2_), 30.5 (CH_2_), 24.6 (Me), 24.4 (Me), 23.3 (Me), 21.2 (Me). GC-MS (EI): *m*/*z* (%) = 266 [M^+^] (1), 236 (50), 224 (8), 207 [M^+^ − AcO] (59), 195 (36), 178 (27), 164 (84), 153 (100), 136 (61), 121 (59), 107 (62), 91 (53), 79 (29).

## 4. Conclusions

Our work provides some relevant findings. First, we demonstrated that fungi are able to perform different biotransformations on the isomeric forms of the C_13_ apocarotenoids ionone, theaspirane and damascone. With respect to the eleven strains tested, we observed that the most common chemical transformations are oxidation reactions that afford oxygenated products such as hydroxy- keto- or epoxy-derivatives. On the contrary, the reduction of the keto groups or the reduction of the double bond functional groups are less relevant transformations, occurring for few substrates and yielding a minority amount of products.

A very significant feature of our study concern the prospective applicability of the fungi-mediated biotransformation of apocarotenoids for the synthesis of high value natural flavours. Since some ionone, damascone and theaspirane isomers are available in natural form and the biotransformation of a natural precursor is considered a ‘natural method’ of synthesis, the flavours obtained by means of the fungi-mediated reactions described above possess the natural status and could be commercialized accordingly. 

Finally, we would like to highlight that our microbial biotransformations allow the preparation of many derivatives whose synthesis, using the classical chemical reactions, is very difficult. For example, different fungal strains proved to be able to oxidize some inactivated positions of the ionone or theaspirane framework, such as the position 2 and 3 of the β- and γ-ionone and the methine carbon linked to the theaspirane oxygen atom. By means of these microbial capabilities, we isolated one new compound (compound **36**) and we devised a new biocatalytic procedure for the synthesis of 2-hydroxy-γ-ionone, 3-hydroxy-γ-ionone, 3,4-dihydroxy-β-ionone and 2,6,10,10-tetramethyl-1-oxa-spiro[4.5]dec-6-ene-2,8-diol (identified as its monoacetate **34**), which are natural apocarotenoids or their direct precursors. 

## Figures and Tables

**Table 1:** Results of the fungi-mediated biotransformation of α-, β- and γ-ionone isomers.

Substrate	Biotransformation Products	Fungal Strains and Distribution of the Biotransformation Products ^1^										
		*A. niger*	*N. oryzae*	*G. candidum*	*Y. lipolytica*	*P. roqueforti*	*R. stolonifer*	*P. corylophilum*	*M. isabellina*	*C. lunata*	*X. dendrorhous*	*F. culmorum*
**α-ionone (1)**	**1**	21	2	100	87	82	85	84	62	-	88	24
	**8**	13	27	-	-	4	2	4	1	2	-	10
	**9a**	33	7	-	1	3	3	4	9	7	2	21
	**9b**	28	25	-	1	10	1	6	12	-	1	25
	**10**	-	-	-	-	-	-	-	-	10	-	3
	**11**	-	-	-	8	-	-	-	3	-	2	-
	**12**	-	10	-	-	-	-	-	-	7	-	2
	N.D. ^2^	5	31	-	3	1	9	2	13	74	7	15
**β-ionone (2)**	**2**	45	15	76	82	87	66	61	84	16	90	40
	**13**	1	-	5	4	1	3	3	1	-	-	4
	**14**	39	26	8	1	8	14	16	7	15	3	11
	**15**	-	-	-	-	-	-	1	-	-	-	-
	**16**	-	-	-	-	-	-	-	-	23	-	3
	**17**	-	26	-	-	-	-	-	-	-	-	-
	**18**	11	-	-	-	-	-	8	-	-	-	-
	**19**	-	-	-	-	2	-	-	-	-	-	-
	**20**	-	-	1	-	-	-	-	-	-	-	-
	**21**	-	2	-	11	-	-	3	1	12	-	3
	**22**	-	5	-	1	-	-	-	-	-	-	-
	N.D. ^2^	4	26	10	1	2	17	8	7	34	7	39
**γ-ionone (3)**	**3**	-	6	100	43	83	100	44	85	26	78	21
	**23a**	35	-	-	-	5	-	14	-	-	-	-
	**23b**	-	-	-	-	-	-	-	-	-	-	-
	**24**	2	-	-	-	-	-	-	2	8	-	13
	**25**	49	4	-	-	-	-	-	-	-	-	10
	**26**	-	2	-	20	-	-	19	-	-	8	7
	**27**	-	2	-	16	1	-	6	2	20	5	-
	N.D. ^2^	14	86	-	21	11	-	17	11	46	9	49

^1^ Percentage of the compound detected by GC-MS analysis of the biotransformation mixture, after extraction and chemical acetylation; ^2^ N.D. = not determined: the value indicates the overall percentage of the compounds obtained by biotransformation whose chemical structure wasn’t assigned.

**Table 2:** Results of the fungi-mediated biotransformation of 3,4-dehydro-β-ionone and theaspirane.

Substrate	Biotransformation Products	Fungal Strains and Distribution of the Biotransformation Products ^1^										
		*A. niger*	*N. oryzae*	*G. candidum*	*Y. lipolytica*	*P. roqueforti*	*R. stolonifer*	*P. corylophilum*	*M. isabellina*	*C. lunata*	*X. dendrorhous*	*F. culmorum*
**3,4-dehydro-β-ionone (4)**	**4**	48	-	45	87	45	80	76	71	52	6	-
	**28**	25	69	18	-	32	7	22	8	26	26	35
	**29**	3	2	17	1	9	3	-	7	8	22	20
	**30**	2	3	12	1	6	2	-	5	6	21	17
	N.D. ^2^	22	26	8	3	8	8	2	9	8	25	28
**theaspirane (5)**	**5**	20	-	56	82	31	-	22	-	8	46	-
	**31**	9	12	11	4	19	5	16	-	-	3	28
	**32**	51	25	15	1	28	62	34	-	3	28	34
	**33**	-	-	-	-	2	-	-	-	-	2	2
	**34**	-	28	-	-	-	6	-	50	8	-	9
	**35**	-	-	-	-	-	-	-	-	12	-	-
	**36**	-	-	-	-	-	-	-	-	9	-	-
	N.D. ^2^	20	35	18	1	20	27	28	50	60	21	27

^1^ Percentage of the compound detected by GC-MS analysis of the biotransformation mixture, after extraction and chemical acetylation; ^2^ N.D. = not determined: the value indicates the overall percentage of the compounds obtained by biotransformation whose chemical structure wasn’t assigned.

**Table 3:** Results of the fungi-mediated biotransformation of α- and β-damascone isomers.

Substrate	Biotransformation Products	Fungal Strains and Distribution of the Biotransformation Products ^1^										
		*A. niger*	*N. oryzae*	*G. candidum*	*Y. lipolytica*	*P. roqueforti*	*R. stolonifer*	*P. corylophilum*	*M. isabellina*	*C. lunata*	*X. dendrorhous*	*F. culmorum*
**α-damascone (6)**	**6**	56	89	55	93	100	100	24	30	96	39	56
	**37**	20	5	10	-	-	-	-	22	-	10	5
	**38**	5	2	10	-	-	-	14	17	3	21	30
	**39**	-		-	-	-	-	-	2	-	3	-
	**40**	-		-	-	-	-	13	-	-	-	-
	N.D. ^2^	19	4	25	7	-	-	49	29	1	27	9
**β-damascone (7)**	**7**	98	70	95	95	100	88	95	95	92	95	57
	**41**	-	2	-	-	-	1	-	-	-	-	4
	**42**	1	23	1	1	-	2	-	2	7	1	22
	N.D. ^2^	1	5	4	4	-	9	5	3	1	4	17

^1^ Percentage of the compound detected by GC-MS analysis of the biotransformation mixture, after extraction and chemical acetylation; ^2^ N.D. = not determined: the value indicates the overall percentage of the compounds obtained by biotransformation whose chemical structure wasn’t assigned.

## Supplementary Materials

The Supplementary Materials are available online.

## References

[1] Walter M.H., Strack D. (2011). Carotenoids and their cleavage products: Biosynthesis and functions. Nat. Prod. Rep..

[2] Serra S. (2015). Recent advances in the synthesis of carotenoid-derived flavours and fragrances. Molecules.

[3] Ma Q.-G., Wang Y.-G., Liu W.-M., Wei R.-R., Yang J.-B., Wang A.-G., Ji T.-F., Tian J., Su Y.-L. (2014). Hepatoprotective sesquiterpenes and rutinosides from *Murraya koenigii* (L.) Spreng. J. Agric. Food Chem..

[4] Schievano E., Morelato E., Facchin C., Mammi S. (2013). Characterization of markers of botanical origin and other compounds extracted from unifloral honeys. J. Agric. Food Chem..

[5] Li C.-Y., Wu T.-S. (2002). Constituents of the stigmas of *Crocus sativus* and their tyrosinase inhibitory activity. J. Nat. Prod..

[6] Ina K., Etō H. (1971). 3-keto-β-ionone in the essential oil from black tea. Agric. Biol. Chem..

[7] Fujimori T., Kasuga R., Matsushita H., Kaneko H., Noguchi M. (1976). Neutral aroma constituents in burley tobacco. Agric. Biol. Chem..

[8] Bolt A.J.N., Purkis S.W., Sadd J.S. (1983). A damascone derivative from *Nicotiana tabacum*. Phytochemistry.

[9] Sato S., Sasakura S., Kobayashi A., Nakatani Y., Yamanishi T. (1970). Flavor of black tea. Part VI. Intermediate and high boiling components of the neutral fraction. Agric. Biol. Chem..

[10] Beekwilder J., van Rossum H.M., Koopman F., Sonntag F., Buchhaupt M., Schrader J., Hall R.D., Bosch D., Pronk J.T., van Maris A.J.A. (2014). Polycistronic expression of a β-carotene biosynthetic pathway in *Saccharomyces cerevisiae* coupled to β-ionone production. J. Biotechnol..

[11] Serra S., Fuganti C., Brenna E. (2005). Biocatalytic preparation of natural flavours and fragrances. Trends Biotechnol..

[12] Prelog V., Meier H.L. (1950). Untersuchungen über organextrakte und harn. 18. Mitteilung. Über die biochemische oxydation von β-jonon im tierkörper. Helv. Chim. Acta.

[13] Mikami Y., Watanabe E., Fukunaga Y., Kisaki T. (1978). Formation of 2S-hydroxy-β-ionone and 4-hydroxy-β-ionone by microbial hydroxylation of β-ionone. Agric. Biol. Chem..

[14] Mikami Y., Fukunaga Y., Arita M., Kisaki T. (1981). Microbial transformation of β-ionone and β-methylionone. Appl. Environ. Microbiol..

[15] Krasnobajew V., Helmlinger D. (1982). Fermentation of fragrances: Biotransformation of β-ionone by *Lasiodiplodia theobromae*. Helv. Chim. Acta.

[16] Hartman D.A., Pontones M.E., Kloss V.F., Curley R.W., Robertson L.W. (1988). Models of retinoid metabolism: Microbial biotransformation of α-ionone and β-ionone. J. Nat. Prod..

[17] Schoch E., Benda I., Schreier P. (1991). Bioconversion of α-damascone by *Botrytis cinerea*. Appl. Environ. Microbiol..

[18] Kakeya H., Sugai T., Ohta H. (1991). Biochemical preparation of optically active 4-hydroxy-β-ionone and its transformation to (*S*)-6-hydroxy-α-ionone. Agric. Biol. Chem..

[19] Weidmann V., Kliewer S., Sick M., Bycinskij S., Kleczka M., Rehbein J., Griesbeck A.G., Zorn H., Maison W. (2015). Studies towards the synthetic applicability of biocatalytic allylic oxidations with the lyophilisate of *Pleurotus sapidus*. J. Mol. Catal. B Enzym..

[20] Gliszczyńska A., Gładkowski W., Dancewicz K., Gabryś B., Szczepanik M. (2016). Transformation of β-damascone to (+)-(*S*)-4-hydroxy-β-damascone by fungal strains and its evaluation as a potential insecticide against aphids *Myzus persicae* and lesser mealworm *Alphitobius diaperinus* Panzer. Catal. Commun..

[21] Lutz-Wahl S., Fischer P., Schmidt-Dannert C., Wohlleben W., Hauer B., Schmid R.D. (1998). Stereo- and regioselective hydroxylation of α-ionone by *Streptomyces* strains. Appl. Environ. Microbiol..

[22] Maurer S.C., Schulze H., Schmid R.D., Urlacher V. (2003). Immobilisation of P450 BM-3 and an NADP+ cofactor recycling system: Towards a technical application of heme-containing monooxygenases in fine chemical synthesis. Adv. Synth. Catal..

[23] Litzenburger M., Bernhardt R. (2016). Selective oxidation of carotenoid-derived aroma compounds by CYP260B1 and CYP267B1 from *Sorangium cellulosum* So ce56. Appl. Microbiol. Biotechnol..

[24] Venkataraman H., Beer S.B.A.d., Geerke D.P., Vermeulen N.P.E., Commandeur J.N.M. (2012). Regio- and stereoselective hydroxylation of optically active α-ionone enantiomers by engineered cytochrome P450 BM3 mutants. Adv. Synth. Catal..

[25] Fuganti C., Serra S., Zenoni A. (2000). Synthesis and olfactory evaluation of (+)- and (-)-gamma-ionone. Helv. Chim. Acta.

[26] Serra S., Fuganti C., Brenna E. (2006). Synthesis, olfactory evaluation, and determination of the absolute configuration of the 3,4-didehydroionone stereoisomers. Helv. Chim. Acta.

[27] Serra S., Fuganti C., Brenna E. (2007). Two easy photochemical methods for the conversion of commercial ionone alpha into regioisomerically enriched gamma-ionone and gamma-dihydroionone. Flavour Fragr. J..

[28] Serra S. (2013). An expedient preparation of enantio-enriched ambergris odorants starting from commercial ionone alpha. Flavour Fragr. J..

[29] Brenna E., Fuganti C., Serra S., Kraft P. (2002). Optically active ionones and derivatives: Preparation and olfactory properties. Eur. J. Org. Chem..

[30] Serra S., Fuganti C. (2006). Synthesis of the enantiomeric forms of alpha- and gamma-damascone starting from commercial racemic alpha-ionone. Tetrahedron Asymmetry.

[31] Brenna E., Fuganti C., Serra S. (2005). Synthesis and olfactory evaluation of the enantiomerically enriched forms of 7,11-epoxymegastigma-5(6)-en-9-one and 7,11-epoxymegastigma-5(6)-en-9-ols isomers, identified in passiflora edulis. Tetrahedron Asymmetry.

[32] Serra S., Barakat A., Fuganti C. (2007). Chemoenzymatic resolution of *cis*- and *trans*-3,6-dihydroxy-alpha-ionone. Synthesis of the enantiomeric forms of dehydrovomifoliol and 8,9-dehydrotheaspirone. Tetrahedron Asymmetry.

[33] Mazur M., Grudniewska A., Wawrzeńczyk C. (2015). Microbial transformations of halolactones with *p*-menthane system. J. Biosci. Bioeng..

[34] Simeo Y., Sinisterra J.V. (2009). Biotransformation of terpenoids: A green alternative for producing molecules with pharmacological activity. Mini-Rev. Org. Chem..

[35] Bhatti H.N., Khera R.A. (2012). Biological transformations of steroidal compounds: A review. Steroids.

[36] Carballeira J.D., Álvarez E., Sinisterra J.V. (2004). Biotransformation of cyclohexanone using immobilized *Geotrichum candidum* NCYC49: Factors affecting the selectivity of the process. J. Mol. Catal. B Enzym..

[37] Bankar A.V., Kumar A.R., Zinjarde S.S. (2009). Environmental and industrial applications of *Yarrowia lipolytica*. Appl. Microbiol. Biotechnol..

[38] Johnson E.A. (2003). *Phaffia rhodozyma*: Colorful odyssey. Int. Microbiol..

[39] Knapp H., Weigand C., Gloser J., Winterhalter P. (1997). 2-hydroxy-2,6,10,10-tetramethyl-1-oxaspiro[4.5]dec-6-en-8-one: Precursor of 8,9-dehydrotheaspirone in white-fleshed nectarines. J. Agric. Food Chem..

[40] Näf R., Velluz A., Decorzant R., Näf F. (1991). Structure and synthesis of two novel ionone-type compounds identified in quince brandy (*Cydonia oblonga* Mil.). Tetrahedron Lett..

[41] Serra S. (2015). MnO_2_/TBHP: A versatile and user-friendly combination of reagents for the oxidation of allylic and benzylic methylene functional groups. Eur. J. Org. Chem..

[42] Serra S., Lissoni V. (2015). First enantioselective synthesis of marine diterpene ambliol-A. Eur. J. Org. Chem..

[43] Dess D.B., Martin J.C. (1983). Readily accessible 12-I-5 oxidant for the conversion of primary and secondary alcohols to aldehydes and ketones. J. Org. Chem..

[44] Audran G., Galano J.M., Monti H. (2001). Enantioselective synthesis and determination of the absolute configuration of natural (−)-elegansidiol. Eur. J. Org. Chem..

[45] Serra S., Gatti F.G., Fuganti C. (2009). Lipase-mediated resolution of the hydroxy-cyclogeraniol isomers: Application to the synthesis of the enantiomers of karahana lactone, karahana ether, crocusatin C and gamma-cyclogeraniol. Tetrahedron Asymmetry.

[46] Kaiser R., Lamparsky D. (1978). Inhaltsstoffe des *Osmanthus*-absolues. 1. Mitteilung: 2,5-epoxy-megastigma-6,8-dien. Helv. Chim. Acta.

[47] Tu V.A., Kaga A., Gericke K.-H., Watanabe N., Narumi T., Toda M., Brueckner B., Baldermann S., Mase N. (2014). Synthesis and characterization of quantum dot nanoparticles bound to the plant volatile precursor of hydroxy-apo-10′-carotenal. J. Org. Chem..

[48] Khachik F., Chang A.-N. (2011). Synthesis of (3*S*)- and (3*R*)-3-hydroxy-β-ionone and their transformation into (3*S*)- and (3*R*)-β-cryptoxanthin. Synthesis.

[49] Takei Y., Mori K., Matsui M. (1973). Synthesis of a stereoisomeric mixture of 3-hydroxy-α-damascone. Agric. Biol. Chem..

[50] Pascual A., Bischofberger N., Frei B., Jeger O. (1988). Photochemical reactions. 149th communication. Photochemistry of 7,8-dihydro-4-hydroxy-β-ionone and derivatives. Helv. Chim. Acta.

[51] Buschor D.J., Eugster C.H. (1990). Synthese der (3*S*,4*R*,3′*S*,4′R)- und (3*S*,4*R*,3′*S*,4′*S*)crustaxanthine sowie weiterer verbindungen mit 3,4-dihydroxy-β-endgruppen. Helv. Chim. Acta.

[52] Irie H., Matsumoto R., Nishimura M., Zhang Y. (1990). Synthesis of (±)-heritol, a sesquiterpene lactone belonging to the aromatic cadinane group. Chem. Pharm. Bull..

[53] Cooper R.D.G., Davis J.B., Leftwick A.P., Price C., Weedon B.C.L. (1975). Carotenoids and related compounds. Part XXXII. Synthesis of astaxanthin, phoenicoxanthin, hydroxyechinenone, and the corresponding diosphenols. J. Chem. Soc. Perkin Trans. 1.

[54] Serra S., Piccioni O. (2015). A new chemo-enzymatic approach to the stereoselective synthesis of the flavors tetrahydroactinidiolide and dihydroactinidiolide. Tetrahedron Asymmetry.

[55] Oritani T., Yamamoto H., Yamashita K. (1990). Synthesis of (±)-4′-hydroxy-γ-ionylideneacetic acids, fungal biosynthetic intermediates of abscisic acid. Agric. Biol. Chem..

