# Supplementary material for: Fungi-Mediated Biotransformation of the Isomeric Forms of the Apocarotenoids Ionone, Damascone and Theaspirane
Source: Molecules. 2018 Dec 21;24(1):19. doi: 10.3390/molecules24010019 (PMC6337586; doi:10.3390/molecules24010019)
Supplement: Supplementary file 1 [file molecules-24-00019-s001.pdf]

Supplementary Materials

Fungi-Mediated Biotransformation of the Isomeric Forms of the Apocarotenoids Ionone, Damascone and Theaspirane

Stefano Serra * and Davide De Simeis

C.N.R. Istituto di Chimica del Riconoscimento Molecolare, Via Mancinelli 7, 20131 Milano, Italy; dav.biotec01@gmail.com

* Correspondence: stefano.serra@cnr.it; stefano.serra@polimi.it; Tel.: +39-02-2399-3076

Characterization data of the compounds used as reference standards in this work

cis-2-Hydroxy-α-ionone acetate = (1SR,5RS)-4,6,6-trimethyl-5-((E)-3-oxobut-1-en-1-yl)cyclohex-3-en-1-yl acetate. ^{1}H-NMR: δ = 6.81 (dd, *J* = 15.9, 10.5 Hz, 1H), 6.08 (d, *J* = 15.9 Hz, 1H), 5.43 (bs, 1H), 4.77 (t, *J* = 4.9 Hz, 1H), 2.46 (d, *J* = 10.5 Hz, 1H), 2.40 (dm, *J* = 18.5 Hz, 1H), 2.27 (s, 3H), 2.15 (d, *J* = 18.5 Hz, 1H), 2.07 (s, 3H), 1.60 (s, 3H), 0.95 (s, 3H), 0.90 (s, 3H); ^{13}C-NMR: δ = 198.1 (C), 170.3 (C), 148.6 (CH), 133.5 (CH), 132.1 (C), 119.1 (CH), 75.1 (CH), 54.2 (CH), 36.2 (C), 28.7 (CH_2), 26.7 (Me), 25.9 (Me), 22.6 (Me), 21.6 (Me), 21.1 (Me); GC-MS (EI): *m/z* (%) = 250 [M^+] (<1), 235 [M^+-Me] (1), 190 (28), 175 (58), 157 (8), 147 (100), 137 (12), 121 (34), 105 (27), 93 (22), 77 (10), 72 (11).

cis-3-Hydroxy-α-ionone acetate (**9a**) = *(1RS,4SR)-3,5,5-trimethyl-4-((E)-3-oxobut-1-en-1-yl)cyclohex-2-en-1-yl acetate.* ^{1}H-NMR: δ = 6.64 (dd, *J* = 15.8, 9.5 Hz, 1H), 6.09 (d, *J* = 15.8 Hz, 1H), 5.53 (s, 1H), 5.36–5.29 (m, 1H), 2.32 (d, *J* = 9.5 Hz, 1H), 2.22 (s, 3H), 2.06 (s, 3H), 1.72 (dd, *J* = 13.0, 6.7 Hz, 1H), 1.64 (m, 3H), 1.51 (dd, *J* = 13.0, 9.4 Hz, 1H), 1.02 (s, 3H), 0.90 (s, 3H); ^{13}C-NMR: δ = 198.1 (C), 170.8 (C), 147.0 (CH), 137.4 (C), 132.8 (CH), 122.1 (CH), 69.4 (CH), 54.1 (CH), 36.3 (CH_2), 34.8 (C), 28.7 (Me), 27.1 (Me), 27.0 (Me), 22.4 (Me), 21.3 (Me).

3-Keto-α-ionol acetate (**12**) = (E)-4-(2,6,6-trimethyl-4-oxocyclohex-2-en-1-yl)but-3-en-2-yl acetate. ^{1}H-NMR: δ = 5.91 (s, 1H), 5.62–5.58 (m, 2H), 5.39–5.29 (m, 1H), 2.59–2.48 (m, 1H), 2.37–2.26 (m, 1H), 2.12–1.98 (m, 1H), 2.04 (s, 3H), 1.89–1.87 (m, 3H), 1.33 and 1.31 (2 d, *J* = 1.6 and 1.6 Hz, 3H), 1.03 (s, 3H), 0.95 (s, 3H); ^{13}C-NMR: δ = 198.9 (C), 170.1 (C), 161.2 (C), 161.2 (C), 133.9 (CH), 133.8 (CH), 129.2 (CH), 129.0 (CH), 126.0 (CH), 126.0 (CH), 70.3 (CH), 55.3 (CH), 55.2 (CH), 47.5 (CH_2), 47.4 (CH_2), 36.1 (C), 36.1 (C), 27.8 (Me), 27.8 (Me), 26.8 (Me), 26.8 (Me), 23.4 (Me), 23.3 (Me), 21.2 (Me), 20.3 (Me), 20.3 (Me); GC-MS (EI): *m/z* (%) = 250 [M^+] (1), 207 (4), 190 (16), 175 (13), 148 (7), 134 (88), 119 (11), 108 (100), 91 (32), 77 (10), 65 (4).

4-Hydroxy-β-ionone acetate (**14**) = *(E)-2,4,4-trimethyl-3-(3-oxobut-1-en-1-yl)cyclohex-2-en-1-yl acetate.* ^{1}H-NMR: δ = 7.18 (d, *J* = 16.4, 1H), 6.13 (d, *J* = 16.4 Hz, 1H), 5.24 (t, *J* = 4.8 Hz, 1H), 2.31 (s, 3H), 2.08 (s, 3H), 1.98–1.42 (m, 4H), 1.70 (s, 3H), 1.09 (s, 3H), 1.05 (s, 3H); ^{13}C-NMR: δ = 198.1 (C), 170.8 (C), 142.0 (CH), 141.6 (C), 133.3 (CH), 129.9 (C), 71.9 (CH), 34.7 (CH_2), 34.5 (C), 28.7 (Me), 27.4 (Me), 27.3 (Me), 25.1 (CH_2), 21.2 (Me), 18.2 (Me); MS (ESI): 273.0 (M^++Na).

4-Keto-β-ionol acetate (**15**) = *(E)-4-(2,6,6-trimethyl-3-oxocyclohex-1-en-1-yl)but-3-en-2-yl acetate,* ^{1}H-NMR: δ = 6.19 (dt, *J* = 16.1, 1.0 Hz, 1H), 5.55 (dd, *J* = 16.1, 6.5 Hz, 1H), 5.44–5.35 (m, 1H), 2.48–2.43 (m, 2H), 2.04 (s, 3H), 1.83–1.78 (m, 2H), 1.74 (d, *J* = 1.0 Hz, 3H), 1.35 (d, *J* = 6.5 Hz, 3H), 1.11 (s, 3H), 1.10 (s, 3H); ^{13}C-NMR: δ = 199.1 (C), 170.1 (C), 159.9 (C), 135.5 (CH), 130.1 (C), 127.7 (CH), 70.7 (CH), 37.2 (CH_2), 35.3 (C), 34.2 (CH_2), 27.1 (Me), 27.1 (Me), 21.2 (Me), 20.3 (Me), 13.2 (Me); GC-MS (EI): *m/z* (%) = 250 [M^+] (13), 208 (100), 190 (12), 175 (26), 165 (80), 147 (24), 134 (59), 119 (32), 105 (22), 91 (24), 77 (13).

4-Hydroxy-β-dihydroionone acetate (**17**) = *2,4,4-trimethyl-3-(3-oxobutyl)cyclohex-2-en-1-yl acetate.* ^{1}H-NMR: δ = 5.13 (t, *J* = 4.5 Hz, 1H), 2.58–2.50 (m, 2H), 2.38–2.24 (m, 2H), 2.15 (s, 3H), 2.06 (s, 3H), 1.98–1.20 (m, 4H), 1.58 (s, 3H), 1.05 (s, 3H), 0.98 (s, 3H); ^{13}C-NMR: δ = 208.1 (C), 171.0 (C), 143.4 (C), 125.8 (C), 72.5 (CH), 43.6 (CH_2), 35.3 (C), 34.7 (CH_2), 29.8 (Me), 28.2 (Me), 26.7 (Me), 25.3 (CH_2), 22.3 (CH_2), 21.4 (Me), 16.4 (Me); MS (ESI): 275.0 (M^++Na).

2-Hydroxy-β-ionone acetate (**18**) = *(E)-2,2,4-trimethyl-3-(3-oxobut-1-en-1-yl)cyclohex-3-en-1-yl acetate.* ^{1}H-NMR: δ = 7.19 (d, *J* = 16.4 Hz, 1H), 6.12 (d, *J* = 16.4 Hz, 1H), 4.79 (dd, *J* = 8.4, 3.2 Hz, 1H), 2.38–1.73 (m, 4H), 2.30 (s, 3H), 2.07 (s, 3H), 1.76 (s, 3H), 1.08 (s, 3H), 1.08 (s, 3H); ^{13}C-NMR: δ = 198.4 (C), 170.8 (C), 142.2 (CH), 134.1 (C), 134.0 (C), 132.6 (CH), 77.2 (CH), 37.7 (C), 30.2 (CH_2), 27.3 (Me), 26.6 (Me), 23.1 (CH_2), 22.8 (Me), 21.4 (Me), 21.2 (Me); GC-MS (EI): *m/z* (%) = 250 [M^+] (2), 235 [M^+ − Me] (29), 190 (7), 175 (100), 157 (13), 147 (31), 133 (10), 119 (8), 105 (13), 91 (9), 77 (5).

3-Hydroxy-β-ionone acetate (**19**) = *(E)-3,5,5-trimethyl-4-(3-oxobut-1-en-1-yl)cyclohex-3-en-1-yl acetate.* ^{1}H-NMR: δ = 7.20 (d, *J* = 16.4 Hz, 1H), 6.12 (d, *J* = 16.4 Hz, 1H), 5.10–5.00 (m, 1H), 2.50 (dd, *J* = 17.6, 5.9 Hz, 1H), 2.30 (s, 3H), 2.15 (dd, *J* = 17.6, 9.0 Hz, 1H), 2.05 (s, 3H), 1.81 (dm, *J* = 12.3 Hz, 1H), 1.76 (s, 3H), 1.60 (t, *J* = 12.0 Hz, 1H), 1.16 (s, 3H), 1.11 (s, 3H); ^{13}C-NMR: δ = 198.2 (C), 170.6 (C), 141.8 (CH), 135.7 (C), 132.5 (CH), 131.3 (C), 67.6 (CH), 43.9 (CH_2), 38.7 (CH_2), 36.4 (C), 29.7 (Me), 28.3 (Me), 27.3 (Me), 21.3 (Me), 21.3 (Me); GC-MS (EI): *m/z* (%) = 250 [M^+] (1), 235 [M^+ − Me] (2), 190 (14), 175 (100), 157 (6), 147 (22), 131 (19), 119 (5), 105 (9), 91 (7), 77 (4).

cis-2-Hydroxy-γ-ionone acetate (**23a**) = *(1SR,3RS)-2,2-dimethyl-4-methylene-3-((E)-3-oxobut-1-en-1-yl)cyclohexyl acetate.* ^{1}H-NMR: δ = 6.97 (dd, *J* = 15.8, 9.9 Hz, 1H), 6.10 (d, *J* = 15.8 Hz, 1H), 4.88 (s, 1H), 4.74 (dd, *J* = 9.2, 4.0 Hz, 1H), 4.61 (s, 1H), 2.65 (d, *J* = 9.9 Hz, 1H), 2.41 (dt, *J* = 14.0, 5.4 Hz, 1H), 2.37–2.05 (m, 1H) 2.28 (s, 3H), 2.07 (s, 3H), 1.93–1.83 (m, 1H), 1.72–1.57 (m, 1H), 0.91 (s, 6H); ^{13}C-NMR: δ = 197.9 (C), 170.4 (C), 146.0 (C), 145.5 (CH), 132.9 (CH) 111.1 (CH_2), 77.8 (CH), 55.9 (CH), 39.0 (C), 31.2 (CH_2), 27.6 (CH_2), 27.3 (Me), 26.2 (Me), 21.1 (Me), 17.8 (Me); GC-MS (EI): *m/z* (%) = 250 [M^+] (12), 235 [M^+ − Me] (1), 208 (13), 190 (45), 175 (40), 165 (36), 147 (100), 131 (23), 122 (39), 109 (96), 91 (34), 79 (35), 71 (12).

3-Hydroxy-γ-ionone acetate (**25**) = *(E)-3,3-dimethyl-5-methylene-4-(3-oxobut-1-en-1-yl)cyclohexyl acetate.* ^{1}H-NMR: δ = 6.82 (dd, *J* = 15.8, 10.1 Hz, 1H), 6.13 (d, *J* = 15.8 Hz, 1H), 4.97 (s, 1H), 4.95–4.86 (m, 1H), 4.63 (s, 1H), 2,73 (dd, *J* = 12.6, 4.9 Hz, 1H), 2.58 (d, *J* = 10.1 Hz, 1H), 2.29 (s, 3H), 2.15–2.01 (m, 1H), 2.03 (s, 3H), 1.85 (dd, *J* = 12.6, 4.5 Hz, 1H), 1.45 (t, *J* = 12.1 Hz, 1H), 0.95 (s, 3H), 0.92 (s, 3H); ^{13}C-NMR: δ = 197.8 (C), 170.3 (C), 145.3 (CH), 144.5 (C), 134.0 (CH), 112.5 (CH_2), 69.8 (CH), 56.0 (CH), 45.5 (CH_2), 41.1 (CH_2), 35.8 (C), 30.4 (Me), 27.3 (Me), 21.4 (Me), 21.3 (Me); GC-MS (EI): *m/z* (%) = 250 [M^+] (<1), 235 [M^+ − Me] (<1), 190 (28), 175 (29), 157 (14), 147 (100), 131 (32), 119 (20), 105 (39), 91 (27), 79 (15), 69 (14), 55 (8).

trans-3,4-Dihydroxy-β-ionone diacetate (**28**) = *(1RS,2RS)-3,5,5-trimethyl-4-((E)-3-oxobut-1-en-1-yl)cyclohex-3-ene-1,2-diyl diacetate.* ^{1}H-NMR: δ = 7.10 (dq, *J* = 16.4, 0.9 Hz, 1H), 6.11 (d, *J* = 16.4 Hz, 1H), 5.51 (d, *J* = 7.8 Hz, 1H), 5.17–5.09 (m, 1H), 2.28 (s, 3H), 2.06 (s, 3H), 2.00 (s, 3H), 1.88–1.66 (m, 2H), 1.62 (br s, 3H), 1.17 (s, 3H), 1.07 (s, 3H); ^{13}C-NMR: δ = 197.8 (C), 170.7 (C), 170.4 (C), 141.0 (CH), 140.2 (C), 133.7 (CH), 128.5 (C), 74.7 (CH), 70.3 (CH), 41.4 (CH_2), 36.4 (C), 29.7 (Me), 27.7 (Me), 27.5 (Me), 21.1 (Me), 20.8 (Me), 16.5 (Me); MS (ESI): 331.2 (M^++Na).

3-Hydroxy-4-keto-β-ionone acetate (**29**) = *(E)-3,5,5-trimethyl-2-oxo-4-(3-oxobut-1-en-1-yl)cyclohex-3-en-1-yl acetate.* ^{1}H-NMR: δ = 7.20 (dq, *J* = 16.5, 1.0 Hz, 1H), 6.22 (d, *J* = 16.5 Hz, 1H), 5.53 (dd, *J* = 13.4, 6.1 Hz, 1H), 2.35 (s, 3H), 2.18 (s, 3H), 2.15–2.01 (m, 2H), 1.84 (d, *J* = 1.0 Hz, 3H), 1.37 (s, 3H), 1.19 (s, 3H); ^{13}C-NMR: δ = 197.0 (C), 193.6 (C), 170.2 (C), 157.1 (C), 139.2 (CH), 134.1 (CH), 130.2 (C), 70.8 (CH), 42.6 (CH_2), 36.9 (C), 30.0 (Me), 28.1 (Me), 25.8 (Me), 20.8 (Me), 13.5 (Me); GC-MS (EI): *m/z* (%) = 264 [M^+] (1), 249 [M^+ − Me] (<1), 221 (8), 204 (4), 194 (7), 176 (83), 161 (29), 151 (14), 136 (100), 121 (33), 105 (7), 91 (16), 77 (8), 65 (6).

3,4-Diketo-β-ionone acetate (**30**) = *(E)-3,3,5-trimethyl-6-oxo-4-(3-oxobut-1-en-1-yl)cyclohexa-1,4-dien-1-yl acetate.* ^{1}H-NMR: δ = 7.29 (dq, *J* = 16.4, 1.0 Hz, 1H), 6.51 (s, 1H), 6.33 (d, *J* = 16.4 Hz, 1H), 2.38 (s, 3H), 2.28 (s, 3H), 1.99 (d, *J* = 1.0 Hz, 3H), 1.35 (s, 6H); ^{13}C-NMR: δ = 197.1 (C), 179.1 (C), 168.5 (C), 154.7 (C), 143.0 (C), 142.3 (CH), 138.4 (CH), 134.6 (CH), 132.7 (C), 39.7 (C), 28.3 (Me), 27.0 (Me), 27.0 (Me), 20.4 (Me), 13.4 (Me); GC-MS (EI): *m/z* (%) = 262 [M^+] (<1), 247 [M^+ − Me] (1), 220 (100), 205 (20), 192 (7), 177 (57), 159 (21), 149 (59), 135 (14), 121 (8), 105 (10), 91 (11), 77 (9).

2-(3-Acetoxy-2,6,6-trimethylcyclohex-1-en-1-yl)ethyl acetate (**35**). ^{1}H-NMR: δ = 5.13 (t, *J* = 4.6 Hz, 1H), 4.05 (dd, *J* = 9.4, 7.5 Hz, 2H), 2.46–2.37 (m, 2H), 2.06 (br s, 6H), 1.92–1.79 (m, 1H), 1.74–1.52 (m, 2H), 1.66 (s, 3H), 1.44–1.33 (m, 1H), 1.08 (s, 3H), 1.00 (s, 3H); ^{13}C-NMR: δ = 171.0 (C), 171.0 (C), 139.5 (C), 128.2 (C), 72.3 (CH), 63.3 (CH_2),

35.1 (C), 34.6 (CH_2), 28.2 (Me), 28.0 (CH_2), 26.8 (Me), 25.3 (CH_2), 21.3 (Me), 21.0 (Me), 16.8 (Me); GC-MS (EI): *m/z* (%) = 268 [M^+] (<1), 226 (13), 208 [M^+ − AcOH] (16), 166 (40), 148 (17), 133 (100), 120 (28), 110 (36), 91 (16), 79 (8).

3,6,6-Trimethyl-1,4,5,6,7,8-hexahydro-3H-3,5a-epoxybenzo[c]oxepin-8-yl acetate (**36**). ^{1}H-NMR: δ = 5.43-5.36 (m, 1H), 5.31 (s, 1H), 4.49 (dt, *J* = 14.2, 2.1 Hz, 1H), 4.20 (d, *J* = 14.2 Hz, 1H), 2.30–1.40 (m, 6H), 2.02 (s, 3H), 1.47 (s, 3H), 1.11 (s, 3H), 1.01 (s, 3H); ^{13}C-NMR: δ = 170.5 (C), 138.7 (C), 116.3 (CH), 105.3 (C), 86.0 (C), 68.4 (CH), 63.7 (CH_2), 40.7 (CH_2), 35.3 (C), 34.3 (CH_2), 30.5 (CH_2), 24.6 (Me), 24.4 (Me), 23.3 (Me), 21.2 (Me); GC-MS (EI): *m/z* (%) = 266 [M^+] (1), 236 (50), 224 (8), 207 [M^+ − AcO] (59), 195 (36), 178 (27), 164 (84), 153 (100), 136 (61), 121 (59), 107 (62), 91 (53), 79 (29).

3-Keto-α-damascone (**37**) = *(E)-4-(but-2-enoyl)-3,5,5-trimethylcyclohex-2-en-1-one.* Colourless solid, m.p. 45 °C (hexane). ^{1}H-NMR: δ = 7.05–6.93 (m, 1H), 6.30 (dq, *J* = 15.5, 1.6 Hz, 1H), 6.01 (s, 1H), 3.39 (s, 1H), 2.68 (d, *J* = 16.6 Hz, 1H), 2.03 (d, *J* = 16.6 Hz, 1H), 1.96 (dd, *J* = 6.9, 1.6 Hz, 3H), 1.86 (d, *J* = 1.1 Hz, 3H), 1.09 (s, 3H), 1.00 (s, 3H). ^{13}C-NMR: δ = 199.1 (C), 197.7 (C), 156.0 (C), 144.8 (CH), 132.4 (CH), 127.6 (CH), 61.7 (CH), 47.0 (CH_2), 36.6 (C), 29.1 (Me), 27.5 (Me), 23.9 (Me), 18.4 (Me); GC-MS (EI): *m/z* (%) = 206 [M^+] (2), 191 [M^+ − Me] (4), 138 (42), 123 (40), 109 (3), 91 (5), 79 (7), 69 (100), 53 (5).

3-Acetoxy-α-damascone (**38**) = *(E)-4-(but-2-enoyl)-3,5,5-trimethylcyclohex-2-en-1-yl acetate.* ^{1}H-NMR: δ = 6.98–6.84 (m, 1H), 6.33 (dq, *J* = 15.5, 1.6 Hz, 1H), 5.62 (s, 1H), 5.39–5.30 (m, 1H), 2.96 (s, 1H), 2.07 (s, 3H), 1.92 (dd, *J* = 6.9, 1.6 Hz, 3H), 1.85 (dd, *J* = 12.6, 9.8 Hz, 1H), 1.65 (dm, *J* = 6.6, 1H),1.62 (m, 3H), 1.03 (s, 3H), 0.90 (s, 3H); ^{13}C-NMR: δ = 200.4 (C), 171.0 (C), 143.2 (CH), 135.5 (C), 132.2 (CH), 123.1 (CH), 69.2 (CH), 60.6 (CH), 36.3 (CH_2), 34.5 (C), 28.3 (Me), 28.3 (Me), 23.0 (Me), 21.3 (Me), 18.2 (Me); GC-MS (EI): *m/z* (%) = 250 [M^+] (2), 235 [M^+ − Me] (<1), 208 (3), 190 (5), 175 (6), 162 (3), 151 (4), 122 (49), 107 (100), 91 (16), 77 (5), 69 (51).

4-Keto-β-damascone (**41**) = *(E)-3-(but-2-enoyl)-2,4,4-trimethylcyclohex-2-en-1-one.* Colourless solid, m.p. 71 °C (hexane). ^{1}H-NMR: δ = 6.81–6.69 (m, 1H), 6,19 (dq, *J* = 16.0, 1.4 Hz, 1H), 2.57 (t, *J* = 6.9 Hz, 2H), 1.97 (dd, *J* = 6.9, 1.4 Hz, 3H), 1.93 (t, *J* = 6.9 Hz, 2H), 1.62 (s, 3H), 1.19 (s, 6H); ^{13}C-NMR: δ = 198.8 (C), 197.8 (C), 160.8 (C), 147.9 (CH), 132.8 (CH), 129.4 (C), 38.0 (CH_2), 34.6 (C), 34.2 (CH_2), 27.2 (Me), 27.2 (Me), 18.6 (Me), 13.0 (Me); GC-MS (EI): *m/z* (%) = 206 [M^+] (36), 191 [M^+ − Me] (39), 178 (4), 163 (16), 150 (13), 138 (37), 121 (15), 109 (9), 91 (8), 79 (10), 69 (100), 55 (7).

4-Acetoxy-β-damascone (**42**) = *(E)-3-(but-2-enoyl)-2,4,4-trimethylcyclohex-2-en-1-yl acetate.* ^{1}H-NMR: δ = 6.77 (dq, *J* = 15.8, 6.9 Hz, 1H), 6.16 (dq, *J* = 15.8, 1.6 Hz, 1H), 5.22 (t, *J* = 5.0 Hz, 1H), 2.08 (s, 3H), 2.04–1.93 (m, 1H), 1.95 (dd, *J* = 6.9, 1.6 Hz, 3H), 1.84–1.73 (m, 1H), 1.69–1.56 (m, 1H), 1.51 (s, 3H), 1.52–1.42 (m, 1H), 1.06 (s, 3H), 1.05 (s, 3H); ^{13}C-NMR: δ = 200.2 (C), 170.8 (C), 146.4 (CH), 145.7 (C), 133.8 (CH), 127.5 (C), 70.9 (CH), 34.8 (CH_2), 33.7 (C), 28.6 (Me), 27.5 (Me), 25.3 (CH_2), 21.2 (Me), 18.4 (Me), 17.7 (Me); GC-MS (EI): *m/z* (%) = 250 [M^+] (13), 235 [M^+ − Me] (<1), 208 (100), 193 (20), 175 (55), 139 (74), 121 (39), 105 (17), 91 (16), 79 (8), 69 (75).
